# New additions to *Ericboehmia* and *Psiloglonium* (*Hysteriaceae*, *Hysteriales*) based on morphology and multilocus phylogeny

**DOI:** 10.3897/mycokeys.138.197356

**Published:** 2026-08-18

**Authors:** Qing Yang, Yu-Lin Ren, Li-Li Liu, Shi-Ping Zou, Kamran Habib, Solomon Boamah, Nalin N. Wijayawardene, Al-Bandari Fahad Al-Arjani, Abdallah M. Elgorban, Faten Zubair Filimban, Ratchadawan Cheewangkoon, Qi-Rui Li

**Affiliations:** 1 State Key Laboratory of Discovery and Utilization of Functional Components in Traditional Chinese Medicine & School of Pharmaceutical Sciences, Guizhou Medical University, Guian New District, Guizhou 561113, China High-Value Food from Mushrooms and Bioactive Plants in the Green Economy Value Chain Research Group, The Institute of Biotechnology and Genetic Engineering, Chulalongkorn University Bangkok Thailand; 2 Department of Entomology and Plant Pathology, Faculty of Agriculture, Chiang Mai University, Chiang Mai 50200, Thailand Center for Yunnan Plateau Biological Resources Protection and Utilization, College of Biological Resource and Food Engineering, Qujing Normal University Qujing China; 3 Center for Yunnan Plateau Biological Resources Protection and Utilization, College of Biological Resource and Food Engineering, Qujing Normal University, Qujing, Yunnan 655011, China Botany and Microbiology Department, College of Science, King Saud University Riyadh Saudi Arabia; 4 High-Value Food from Mushrooms and Bioactive Plants in the Green Economy Value Chain Research Group, The Institute of Biotechnology and Genetic Engineering, Chulalongkorn University , Bangkok, Thailand Center of Excellence in Biotechnology Research (CEBR), DSR, King Saud University Riyadh Saudi Arabia; 5 Botany and Microbiology Department, College of Science, King Saud University, Riyadh 11451, Saudi Arabia Division of Plant Sciences, Department of Biological Sciences, Faculty of Sciences, King Abdulaziz University Jeddah Saudi Arabia; 6 Center of Excellence in Biotechnology Research (CEBR), DSR, King Saud University, Riyadh, Saudi Arabia State Key Laboratory of Discovery and Utilization of Functional Components in Traditional Chinese Medicine & School of Pharmaceutical Sciences, Guizhou Medical University Guizhou China; 7 Division of Plant Sciences, Department of Biological Sciences, Faculty of Sciences, King Abdulaziz University, Jeddah, Saudi Arabia Department of Entomology and Plant Pathology, Faculty of Agriculture, Chiang Mai University Chiang Mai Thailand

**Keywords:** Karst ecosystem, Saprobes, Southwestern China, Taxonomy, Woody substrates

## Abstract

*Hysteriaceae* is a taxonomically diverse family of ascomycetes, and continued taxonomic studies are improving our understanding of its species diversity and phylogenetic relationships. Two new species of *Hysteriaceae*, *Ericboehmia guizhouensis***sp. nov**., and *Psiloglonium yunnanense***sp. nov**., are described from southwestern China, delineated through an analysis of both morpho-anatomical characteristics and multilocus phylogenetic analyses based on ITS, LSU, SSU, *tef*1-α, and *rpb2* sequence data. Both species share key morphological characteristics typical of *Hysteriaceae*, while their differentiation from previously described congeners was substantiated by comparative morphological characters and phylogenetic analyses. Comprehensive morphological descriptions, detailed illustrations, and a multilocus phylogenetic analysis demonstrating the phylogenetic placements of the new taxa presented. Furthermore, SSU and *tef*1-α sequence data from additional collections of *Psiloglonium macrosporum*, are provided in this study. These findings expand the known diversity of *Ericboehmia* and *Psiloglonium* and enhance our understanding of their taxonomic framework and evolutionary relationships based on morphology and multilocus phylogeny.

## Introduction

The family *Hysteriaceae* was established by Chevallier (1826) and currently comprises 14 genera (Wijayawardene et al. 2022; Hyde et al. 2024). Taxa in this family are predominantly saprobic, occurring on dead plant substrates (Hyde et al. 2020). Morphologically, its members are characterized by exhibiting superficial, typically hysterothecioid or navicular ascomata occasionally branched or discoid; hamathecium with pseudoparaphyses, sometimes darkened or branched apices above the asci; clavate to cylindrical, fissitunicate asci with a distinct apical chamber; ellipsoid, fusiform to clavate, hyaline to brown, variably septate ascospores with smooth or ornamented walls and sometimes surrounded by a sheath; as well as pycnidial or hyphomycetous asexual morphs (Jaklitsch et al. 2016; Jayasiri et al. 2018). The present study focuses on studying two genera within this family, viz., *Ericboehmia* and *Psiloglonium*, and introduces two new species, one in each genus, from southwestern China.

*Ericboehmia* was introduced into *Hysteriaceae* by Gardiennet et al. (2019), with *E. saulensis* as the type species. Following the establishment of *Ericboehmia* subsequent taxonomic revisions were provided by Gardiennet et al. (2019). Eight species are currently accepted in the genus according to Species Fungorum (Species Fungorum 2026, accessed on 17^th^ July 2026). The genus is characterized by having superficial to erumpent ascomata, laterally flattened with a convex ridge and longitudinal slit, carbonaceous peridium, hamathecium with numerous, trabeculate pseudoparaphyses in a gelatinous matrix, bitunicate, 8-ascospored, clavate asci with an ocular chamber; hyaline to pale brown, smooth-walled, initially 1-septate ascospores constricted at the median septum, later developing secondary distosepta and occasionally with longitudinal pseudosepta and polar appendages (Boehm et al. 2009a, 2009b). The genus is readily distinguished from the morphologically similar *Ostreichnion* by its two-celled ascospores (with occasional secondary distosepta) and distant phylogenetic placement (Gardiennet et al 2019).

*Psiloglonium* was established by von Höhnel (1918), although a type species was not designated at that time. Later, Petrak (1923a) designated *P. lineare* as the type species and circumscribed the genus to accommodate didymospored taxa segregated from *Glonium*. Zogg (1962) synonymized *Psiloglonium* under *Glonium*, a decision supported by von Arx and Müller (1975). However, the taxonomic boundaries between *Glonium* and *Psiloglonium* remained controversial for decades until Boehm et al. (2009a) reinstated *Psiloglonium* within *Hysteriaceae*, based on morphological and molecular analyses (von Höhnel 1918; Petrak 1923a, 1923b; Zogg 1962; von Arx and Müller 1975). Currently, 19 species are listed in Species Fungorum (Species Fungorum 2026, accessed on 6^th^ July 2026) under this genus, of which ten have sequence data available in GenBank. The sexual morph of the genus is characterized by having dark, superficial hysterothecial ascomata, bitunicate, fissitunicate, clavate asci containing four to eight ascospores that are fusiform, septate, hyaline to brown and may be surrounded by a sheath. The sporidesmium-like asexual morph has been reported (Rossman et al. 2016). Species of *Psiloglonium* have a broad geographical distribution, with records from China, Thailand, the USA, Kenya, and South Africa, and are primarily reported as saprobes on members of *Caprifoliaceae*, *Ephedraceae*, *Loganiaceae*, and *Poaceae* hosts (Boehm et al. 2009a; Liu et al. 2015; Li et al. 2016).

During a survey of hysteriaceous fungi in southwestern China, three taxa belonging to *Ericboehmia* and *Psiloglonium* were collected. Morphological comparisons, together with phylogenetic analyses based on sequence data of the internal transcribed spacer region (ITS), the large subunit (LSU) and ribosomal small subunit (SSU), the translation elongation factor 1-alpha (*tef*1-α), and the second largest subunit of RNA polymerase II (*rpb*2), showed that two taxa did not correspond to any previously described species within their respective genera and the other species was identified as *Psiloglonium macrosporum*. Furthermore, newly generated SSU, *tef*1-α sequences from additional collections of *Psiloglonium macrosporum* are presented to improve the molecular dataset available for this species. Accordingly, we introduce two new species and provide detailed diagnoses, comprehensive descriptions, illustrations, and a phylogenetic tree to support their taxonomic placements.

## Materials and methods

### Sample collection

Fungal specimens were collected during field surveys in the karst landscapes of Guizhou and Yunnan provinces, in southwestern China, between August and November 2024, primarily targeting saprobic fungi inhabiting dead twigs. All relevant habitat information (hosts, collection date, collector, altitude, climatic conditions, and geographical features) was recorded in a detailed manner. Specimens were placed in paper bags and taken to the laboratory for morphological examination and isolation (Liu et al. 2025).

### Isolation and morphological characterization

Morphological characteristics of the stromata (ostiole, clypeus, etc.) were examined with an Olympus SZ61 stereomicroscope (Japan) and photographed with a Canon 700D digital camera (Canon, Tokyo, Japan). The morphological characters such as size, color, thickness, structure, and texture of the stromata were measured and documented. The specimens were carefully hand sectioned using a sharp blade, and images of the internal structures and morphological characteristics of the ascomata were captured with a Canon digital camera (Canon 700D, Tokyo, Japan). Microscopic morphological characters (ascomata, peridium, paraphyses, asci, ascospore, etc.) were observed using an optical microscope (Nikon Ni, Tokyo, Japan) and photographed using a Canon 700D digital camera attached, and photomicrographs were taken as outlined in Long et al. (2019). For anatomical inspection, specimens were mounted in water and Melzer’s iodine reagent was used to test the apical apparatus structures for amyloid reaction. At least 25 ascospores and 25 asci were measured with Tarosoft (R) Image frame Work (v. 0.9.0.7; Tarosoft, Thailand). Adobe Photoshop CS6 (Adobe Systems, USA) was used to arrange the images. Pure cultures were obtained via single-ascospore isolation following Habib et al. (2025) and maintained at 25 °C on potato dextrose agar (PDA).

### DNA extraction, Polymerase Chain Reaction (PCR) amplification and sequencing

Mycelium grown on pure culture media for 5 weeks was scraped from the culture plate using a sterile scalpel, and genomic DNA was extracted following the protocol of the Solarbio Fungal Genomic DNA Extraction Kit (Beijing Solarbio Science & Technology Co., Ltd., China). For specimens without germinating ascospores, we extracted the DNA directly from the contents of the perithecium. All DNA samples were stored at -20 °C for long term usage. Five gene regions were amplified for phylogenetic analysis: internal transcribed spacers (ITS), partial 18S small subunit rDNA (SSU), partial large subunit rDNA (LSU), partial translation elongation factor-1α (*tef*1-α), and partial RNA polymerase II second largest subunit II (*rpb*2), using the primers ITS1/ITS4 (White et al. 1990, Gardes and Bruns 1993), NS1/NS4 (White et al.1990), LR0R/ LR5 (Vilgalys and Hester 1990), EF1-983F/EF1-2218R (Carbone and Kohn 1999) and rpb2-5f/rpb2-7cr (Liu et al. 1999) respectively. The components of a 25 μL PCR mixture were 9.5 μL of double-distilled water, 12.5 μL of PCR Master Mix, 1 μL of each primer, and 1 μL of template DNA. The PCR amplification was conducted as reported by Samarakoon et al. (2022). Amplification products were visualized on 1.5% agarose gel electrophoresis stained with GoldenView. Qualified PCR products were purified and sequenced by Sangon Biotech (Shanghai, China) (Liu et al. 2025).

### Sequence alignments and phylogenetic analyses

Sequences were compared with existing sequences in GenBank using the BLASTN algorithm to identify similar sequences. Phylogenetic analyses were based on a concatenated dataset of ITS, LSU, SSU, *tef*1-α and *rpb*2 regions. Reference sequences were retrieved from public databases based on recent publications (e.g. Zhang et al. 2023; Raymundo et al. 2024; Sun et al. 2025) and BLASTN results of closely related taxa. Alignments were performed using MAFFT v.7.110 (Katoh et al. 2019) with default parameters and manually refined in BioEdit v.7.0.5.3 (Hall 1999) where necessary. Maximum likelihood (ML) analyses were performed using RAxML-HPC BlackBox v.8.2.12 under the GTRGAMMA substitution model with 1,000 rapid bootstrap replicates (Stamatakis 2014). Bayesian inference (BI) analyses were performed using MrBayes on ACCESS v.3.2.7a (Ronquist et al. 2012) through the CIPRES Science Gateway v.3.3 (Miller et al. 2010). Posterior probabilities (PP) were estimated using the Markov chain Monte Carlo (MCMC) algorithm. Six simultaneous Markov chains were run for 1,000,000 generations, sampling every 1,000^th^ generation. The first 25% of the trees were discarded as burn-ins. The remainder was used to calculate the posterior probabilities (PPs) for individual branches. Resulting phylogenetic trees were visualized in FigTree v.1.4.4 (Rambaut 2018). All sequences generated in this study were deposited in GenBank (Table 1).

### Depositing specimens and cultures

Voucher specimens were deposited at the Herbarium of Guizhou Medical University (**GMB**) and the Herbarium of Cryptogams, Kunming Institute of Botany, Chinese Academy of Sciences (**KUN-HKAS**), and living cultures were deposited at the Guizhou Medical University Culture Collection (**GMBC**). All scientiﬁc names of fungi follow the entries in MycoBank and Index Fungorum, hence no authorities and years of publications are given in the text apart from the taxonomic entries.

### Registration of novel taxa

Index Fungorum Registration Identifiers were obtained for the new species as mentioned in Index Fungorum (2026).

## Results

### Phylogenetic analyses

**Figure 1.**
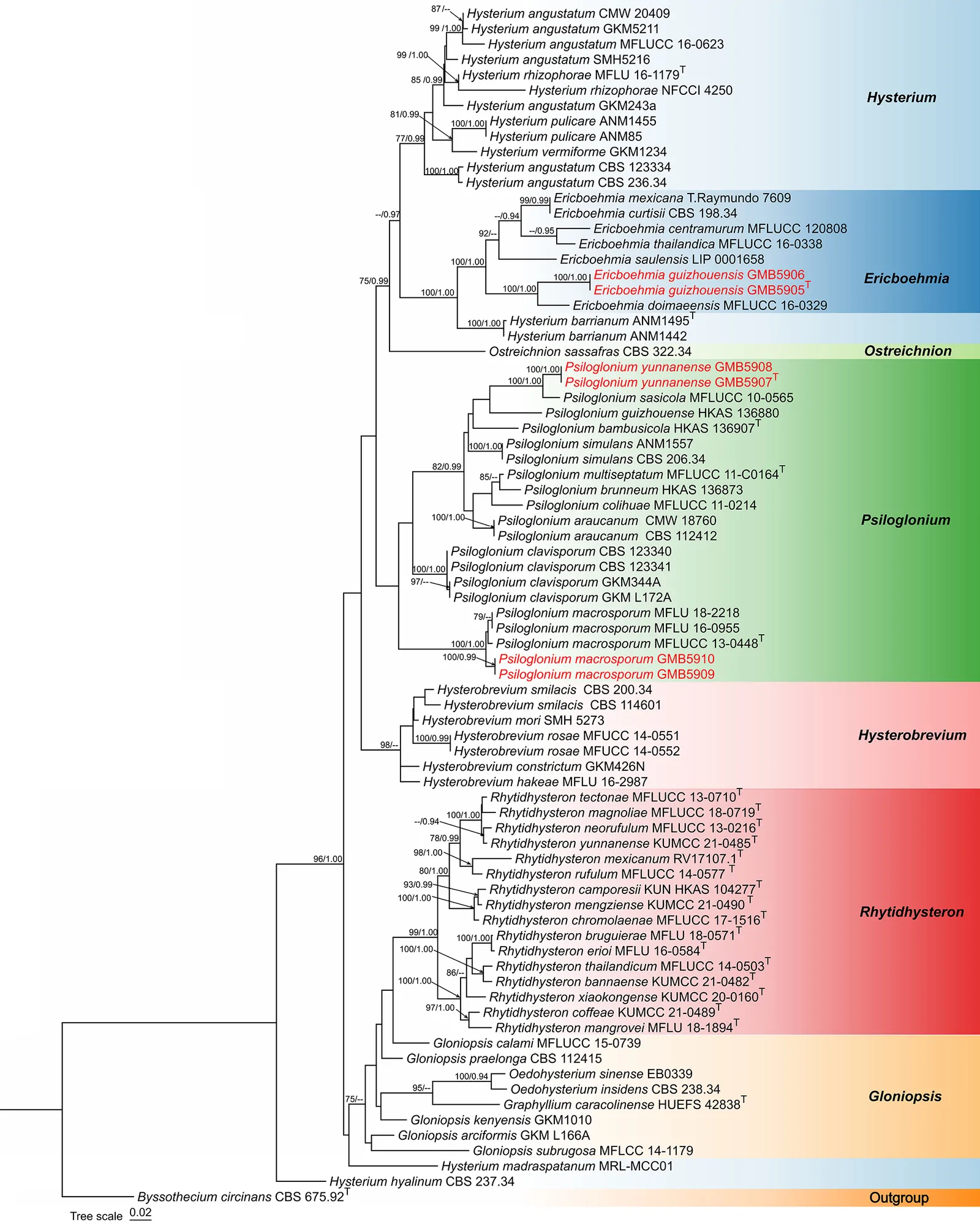
RAxML tree of *Hysteriaceae* based on a combined ITS, LSU, SSU, *rpb*2, and *tef*1-α sequences dataset. *Byssothecium circinans* (CBS 675.92) was used as the outgroup taxon. Bootstrap values for maximum likelihood (left) of ≥70% and Bayesian posterior probabilities (right) of ≥ 0.90 are given near the nodes. The newly isolated strains are indicated in red. Ex-type/type strains are indicated with “^T^” after the strain number.

The combined ITS-LSU-SSU*-rpb*2-*tef*1-α sequence matrix comprised 78 strains, including the newly described species. *Byssothecium circinans* (CBS 675.92) was added as the outgroup taxon. After the exclusion of ambiguously aligned regions and long gaps, the final combined data matrix contained 4,573 characters (ITS:1–559, LSU:560–1,485 bp, SSU: 1,486–2,526 bp, *tef*1-α: 2,527–3,755 bp, *rpb*2: 3,578–4,573 bp). Maximum likelihood analyses with 1,000 bootstrap replicates yielded the best-scoring tree (lnL = -28,902.282), with model parameters alpha: 0.504522; base frequencies A = 0.244333, C = 0.247014, G = 0.278169, T = 0.230484 with the following substitution rates AC = 1.243967, AG = 3.091085, AT = 1.256216, CG = 0.877799, CT = 7.992653, GT = 1.000000. Tree topology from ML analysis was similar to BI analysis. The best-scoring RAxML tree is shown in Fig. 1.

In the phylogram, the newly generated sequences of *Ericboehmia guizhouensis* (GMB5905, GMB5906) formed a well-supported sister clade to *E. doimaeensis* (MFLUCC 16-0329) with 100%/1.00, ML/BI support values (Fig. 1). The sequences of *Psiloglonium macrosporum* (GMB5909, GMB5910) clustered with the reference strains of *P. macrosporum* (MFLUCC 13-0448, MFLU 16-0955, MFLU 18-2218) with maximum support values (100%/0.99; ML/BI). In contrast, the newly generated sequences of *P. yunnanense* (GMB5907, GMB5908) formed a distinct, well-supported sister clade to *P. sasicola* (MFLUCC 10-0565) with 100/1.00 maximum likelihood and statistical support respectively.

### Taxonomy

#### *Dothideomycetes* O.E. Erikss. & Winka

***Hysteriales* Lindau**

***Hysteriaceae* Chevall**

***Ericboehmia* Gardiennet, Lechat & J. Fourn**.

##### Ericboehmia guizhouensis

Taxon classificationFungiHysterialesHysteriaceae

Q. Yang, Q.R. Li & Cheewangkoon sp. nov.

A4C57422-E2D6-53EC-AF6B-C6A0F653CC44

Fig. 2

###### Etymology.

The specific epithet “guizhouensis” refers to the geographical location, Guizhou Province, where the holotype specimen was collected.

###### Type.

China • Guizhou Province, Tongren City, Foding Mountain Forest Nature Reserve (27°19'40.64"N, 108°8'37.63"E), altitude: 701 m, on dead twigs of an unidentified host, 8 August 2024, Qing Yang, 2024FDSDCY41 (holotype; GMB5905; ex-type living culture GMBC5905); *ibid*., isotype: KUN-HKAS151615).

###### Description.

Saprobic on dead twigs. ***Sexual morph*: *Hysterothecia*** 448–987 µm long, 273–455 µm wide, 230–365 µm high (x̄= 671 × 351 × 300 μm, n = 15), scattered, superficial, conchate, narrowing at base, black, shiny, smooth to slightly striate along the length, with a longitudinal slit on upper ridge. ***Peridium*** 39–65 µm wide (x̄ = 51.7 µm, n = 10), carbonaceous, brittle, heavily pigmented, cells prosenchymatous. ***Periphyses*** 0.8–1.5 µm wide, numerous, along the slit, aseptate, hyaline, swollen, with blunt ends. ***Hamathecium*** consisting of numerous, persistent, trabeculate pseudoparaphyses, hyaline, aseptate, branched, borne in a gel matrix, longer than asci. ***Asci*** 84–105 × 13–18.8 µm (x̄ = 96.7 × 15.6 µm, n = 30), 8-spored, bitunicate, oblong to clavate, with a short, rounded pedicel, with an ocular chamber. ***Ascospores*** 22–29 × 5.9–8.2 µm (x̄ = 26.2 × 7.3 µm, n = 30), biseriate, fusiform when young, oblong at maturity, hyaline to light yellow, 1-euseptate at median to submedian, smooth-walled, with 1–2-secondary distosepta, guttulate, lacking mucilaginous sheath and poler appendages. ***Asexual morph***: Undetermined.

**Figure 2.**
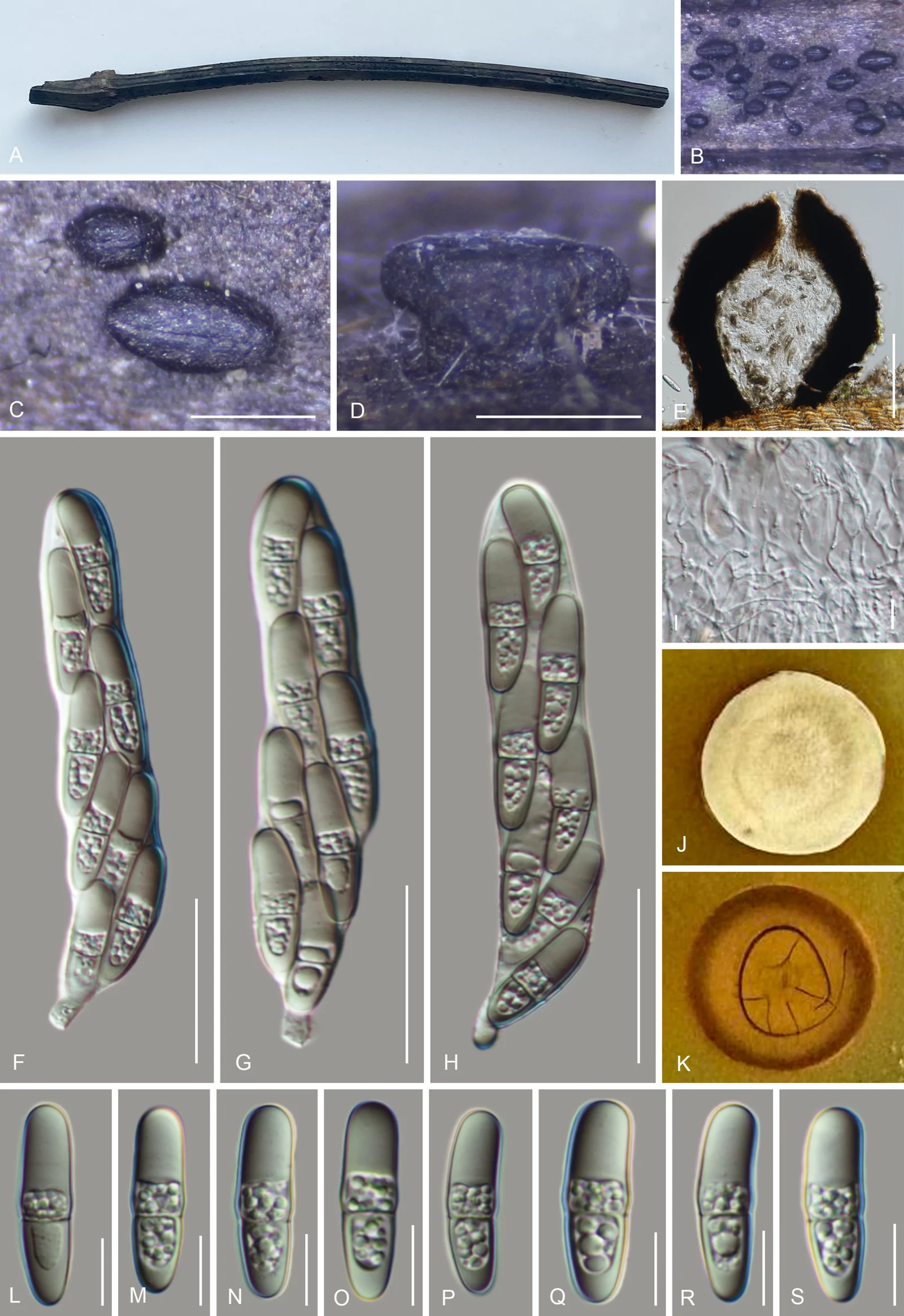
*Ericboehmia guizhouensis* (GMB5905, holotype). **A**. Host substrate; **B–D**. Hysterothecia on host surface; **E**. Hand section of an ehysterothecium; **F–H**. Asci with ascospores; **I**. Hamathecium; **J**. Upper view of colony on PDA; **K**. Reverse view of colony on PDA; **L–S**. Ascospores. Scale bars: 0.5 mm **(B–D)**; 100 μm **(E)**; 30 μm **(F–H)**; 10 μm **(I, L–S)**.

###### Culture characteristics.

Ascospores germinating on PDA within 24 hours. Colonies on PDA reaching 20–30 mm after 5 weeks (day–night cycle) at 25 °C, slow growing, circular, grayish white, centrally raised, compact, with short aerial mycelium and entire margins. Colony surface white to grayish white; reverse dark brown at the margin and orange-yellow in the center. Yellowish brown exudates were released to the media when mycelium growing.

###### Paratype.

China • Guizhou Province, Tongren City, Foding Mountain Forest Nature Reserve (27°19'46.72"N, 108°8'11.55"E), altitude: 874 m, on dead twigs of an unidentified host, 8 August 2024, Qing Yang, 2024FDSDCY78 (GMB5906; GMBC5906).

###### Notes.

Phylogenetically, *Ericboehmia guizhouensis* is closely related to *E. doimaeensis* (MFLUCC 16-0329), forming a sister species relationship (100/1.00; ML/BI, Fig. 1). The LSU sequence of strain *E. guizhouensis* differs from that of strain *E. doimaeensis* (MFLUCC 16-0329) by 32 base pairs out of 857 bp (3.73%, 8/857 including gaps), while the SSU sequence differs by 19 base pairs out of 603 bp (3.15%, 1/603 including gaps). However, *E. guizhouensis* can be distinguished morphologically from *E. doimaeensis* by its smaller hysterothecia (448–987 × 273–455 × 230–365 μm, x̄ = 671 × 351 × 300 μm vs. 600–800 × 380–420 × 480–550 μm, x̄ = 700 × 400 × 510 μm), distinctly smaller asci (84–105 × 13–18.8 µm, x̄ = 96.7 × 15.6 µm vs. 139–225 × 22–44 µm, x̄ = 180 × 36 µm), and ascospores (22–29 × 5.9–8.2 µm, x̄ = 26.2 × 7.3 µm vs. 60–73 × 12–13 µm, x̄ = 66 × 12.5 µm) (Jayasiri et al. 2018). *Ericboehmia guizhouensis* can be readily distinguished from all other species of the genus by its shorter asci and ascospores. Therefore, based on the above morphological differences and phylogeny, we introduce *E. guizhouensis* as a new species.

#### *Psiloglonium* Höhn.

##### Psiloglonium macrosporum

Taxon classificationFungiMytilinidialesGloniaceae

Thambug., Senan. & K.D. Hyde, Fungal Diversity 78: 26 (2016)

ED174B06-018C-5BD4-8C72-059B9C8AB546

###### Material examined.

China • Yannan Province, Ailaoshan (24°5'7.01"N, 101°31'30.44"E), altitude: 1169 m, on decaying wood, 15 October 2024, Qing Yang 2024ALS232 (GMB5909 and GMB5910).

###### Description.

***Sexual morph***: see Li et al. (2016); Jayasiri et al. (2018); Zhang et al. (2023). ***Asexual morph***: undetermined.

###### Notes.

*Psiloglonium macrosporum* was previously known from northern Thailand (Li et al. 2016; Jayasiri et al. 2018) and Guizhou Province, China (Zhang et al. 2023). The present collection from Ailaoshan, Yunnan Province, represents a new provincial record and further extends the known distribution of the species in southwestern China. Our new collection (GMB5909, GMB5910) clustered with *P. macrosporum* with strong statistical support (ML 100% and BI 0.99) in the phylogenetic analysis (Fig. 1). The morphology of *P. macrosporum* (GMB5909) agrees well with previous descriptions of the species in having elongate, hysterothecial ascomata, carbonaceous peridium, trabeculate pseudoparaphyses, 4-spored, bitunicate asci, and large, muriform, brown ascospores surrounded by a mucilaginous sheath. Compared with the ex-type strain (MFLUCC 13-0448), GMB5909 differs in having 4-spored rather than 8-spored asci. It is morphologically similar to the collection reported by Jayasiri et al. (2018), but possesses slightly longer asci (146–216 × 46–69 μm, x̄ = 194 × 58 μm vs. 110–145 × 28–35 μm, x̄ = 130 × 32 μm) and somewhat larger ascospores (79–100 × 24–30 μm, x̄ = 84 × 27 μm vs. 50–100 × 17–24 μm, x̄ = 78 × 22 μm). In contrast to the collection described by Zhang et al. (2023), GMB5909 has muriform, multiseptate ascospores at maturity, whereas the latter was reported to have predominantly two-celled ascospores. These differences may represent intraspecific variation associated with the considerable geographical separation between the collection sites, although variation resulting from different developmental stages cannot be excluded. Overall, GMB5909 conforms well to the circumscription of *P. macrosporum*. The type species *P. macrosporum* (MFLUCC 13-0448) has only ITS and LSU data available; we here supplemented molecular dataset with additional sequences, including SSU and *tef*1-α for the species of *P. macrosporum*.

##### Psiloglonium yunnanense

Taxon classificationFungiMytilinidialesGloniaceae

Q. Yang, Q. R. Li & Cheewangkoon sp. nov.

69E8D452-3733-52CF-936E-AE7BED034D4D

Fig. 3

###### Etymology.

The specific epithet “ *yunnanense* “ refers to the geographical location, Yunnan Province, where the holotype specimen was collected.

**Table 1.** GenBank accession numbers used in this study.

**Species name**	**Strain**	**GenBank accession numbers**					**References**
		**ITS**	**LSU**	**SSU**	***tef*1-α**	***rpb*2**	
*Byssothecium circinans*	CBS 675.92	OM337536	AY016357	AY016339	GU349061	DQ767646	Schoch et al. (2009)
*Ericboehmia centramurum*	MFLUCC 120808	KM272258	KM272256	KM272257	KM277819	NA	Tibpromma et al. (2017)
*Ericboehmia curtisii*	CBS:198.34	NA	MH866967	NA	FJ161093	NA	Barr (1975)
*Ericboehmia doimaeensis*	MFLUCC 16-0329	NA	MH535894	MH535886	NA	NA	Jayasiri et al. (2018)
***Ericboehmia guizhouensis***	GMB5905^T^	PZ281536	PZ332761	PZ513294	PZ528904	PZ544644	This study
***Ericboehmia guizhouensis***	GMB5906	PZ281537	PZ332762	PZ513295	PZ529488	PZ544645	This study
*Ericboehmia mexicana*	T.Raymundo 7609	NA	PP575996	NA	NA	NA	Raymundo et al. (2024)
*Ericboehmia saulensis*	LIP 0001658	NA	MN338581	NA	NA	NA	Gardiennet et al. (2019)
*Ericboehmia thailandica*	MFLUCC 16-0338	MH535873	MH535895	NA	NA	MH535876	Jayasiri et al. (2018)
*Gloniopsis arciformis*	GKM L166A	NA	GU323211	GU323180	NA	NA	Schoch et al. (2009)
*Gloniopsis calami*	MFLUCC15-0739	KX669036	NG059715	KX669034	KX671965	NA	Hyde et al. (2016)
*Gloniopsis kenyensis*	GKM1010	NA	GQ221891	NA	NA	NA	Mugambi and Huhndorf (2009)
*Gloniopsis praelonga*	CBS112415	NA	FJ161173	FJ161134	FJ161090	NA	Boehm et al. (2009a)
*Gloniopsis subrugosa*	MFLCC 14-1179	MH535871	MH535892	MH535884	NA	MH535874	Jayasiri et al. (2018)
*Graphyllium caracolinens*e	HUEFS 42838^T^	NA	KF914914	NA	NA	NA	Almeida et al. (2014)
*Hysterium angustatum*	GKM5211	NA	GQ221906	NA	NA	NA	Mugambi and Huhndorf (2009)
*Hysterium angustatum*	CMW 20409	NA	FJ161194	FJ161153	NA	NA	Boehm et al. (2009b)
*Hysterium angustatum*	SMH5216	NA	GQ221908	NA	GQ221933	NA	Mugambi and Huhndorf (2009)
*Hysterium angustatum*	GKM243a	NA	GQ221899	NA	GQ221928	NA	Mugambi and Huhndorf (2009)
*Hysterium angustatum*	CBS 123334	NA	FJ161207	NA	NA	NA	Boehm et al. (2009b)
*Hysterium angustatum*	CBS:236.34	NA	FJ161180	GU397359	FJ161096	FJ161117	Boehm et al. (2009b)
*Hysterium angustatum*	MFLUCC 16-0623	NA	MH535893	MH535885	MH535878	MH535875	Jayasiri et al. (2018)
*Hysterium barrianum*	ANM1495	NA	GQ221885	NA	GQ221931	NA	Mugambi and Huhndorf (2009)
*Hysterium barrianum*	ANM1442	NA	GQ221884	GU323181	NA	NA	Mugambi and Huhndorf (2009)
*Hysterium hyalinum*	CBS:237.34	NA	FJ161181	FJ161141	NA	NA	Boehm et al. (2009b)
*Hysterium madraspatanum*	MRL MCC01	OR420067	NA	NA	NA	NA	Manogaran et al. (2024)
*Hysterium pulicare*	ANM85	NA	GQ221898	NA	GQ221934	NA	Mugambi and Huhndorf (2009)
*Hysterium pulicare*	ANM1455	NA	GQ221904	NA	GQ221932	NA	Mugambi and Huhndorf (2009)
*Hysterium rhizophorae*	MFLU 16-1179^T^	KX611363	KX611364	KX611365	NA	NA	Hyde et al. (2017)
*Hysterium rhizophorae*	NFCCI 4250	MG844284	MG844276	MG844280	NA	MG968956	Devadatha et al. (2018)
*Hysterium vermiforme*	GKM1234	NA	GQ221897	NA	GQ221929	NA	Mugambi and Huhndorf (2009)
*Hysterobrevium constrictum*	GKM 426N	NA	GQ221901	NA	GQ221913	NA	Mugambi and Huhndorf (2009)
*Hysterobrevium hakeae*	MFLU 16-2987	NA	MH535896	NA	NA	NA	Jayasiri et al. (2018)
*Hysterobrevium mori*	SMH 5273	NA	GU301820	NA	NA	NA	Boehm et al. (2009b)
*Hysterobrevium rosae*	MFUCC 14-0551	NA	MH535897	NA	MH535879	NA	Jayasiri et al. (2018)
*Hysterobrevium rosae*	MFUCC 14-0552	NA	MH535898	MH535887	MH535880	NA	Jayasiri et al. (2018)
*Hysterobrevium smilacis*	CBS 114601	NA	FJ161174	FJ161135	FJ161091	FJ161114	Boehm et al. (2009b)
*Hysterobrevium smilacis*	CBS 200.34	NA	FJ161177	FJ161138	NA	NA	Boehm et al. (2009b)
*Oedohysterium insidens*	CBS:238.34	NA	FJ161182	FJ161142	FJ161097	NA	Boehm et al. (2009b)
*Oedohysterium sinense*	EB0339	NA	GU397348	GU397364	GU397339	GU397357	Boehm et al. (2009a)
*Ostreichnion sassafras*	CBS:322.34	MH855548	MH867054	FJ161148	NA	FJ161122	Vu et al. (2019)
*Psiloglonium araucanum*	CMW 18760	NA	FJ161192	FJ161151	NA	NA	Boehm et al. (2009a)
*Psiloglonium araucanum*	CBS 112412	NA	FJ161172	FJ161133	FJ161089	FJ161112	Boehm et al. (2009a)
*Psiloglonium bambusicola*	HKAS 136907	NA	PQ644554	PQ651963	PQ761116	NA	Sun et al. (2025)
*Psiloglonium brunneum*	HKAS 136873	NA	PQ644557	PQ651964	PQ761118	NA	Sun et al. (2025)
*Psiloglonium clavisporum*	CBS 123340	NA	FJ161205	NA	NA	NA	Boehm et al. (2009a)
*Psiloglonium clavisporum*	CBS 123341	NA	FJ161206	NA	NA	NA	Boehm et al. (2009a)
*Psiloglonium clavisporum*	GKM L172A	NA	GU323204	GU323192	NA	NA	Schoch et al. (2009)
*Psiloglonium clavisporum*	GKM344A	NA	GQ221889	NA	NA	NA	Mugambi and Huhndorf (2009)
*Psiloglonium colihuae*	MFLUCC 11-0124	NA	KP744511	NA	NA	NA	Liu et al. (2015)
*Psiloglonium guizhouense*	HKAS 136880	PQ637064	PQ644556	NA	PQ761117	NA	Sun et al. (2025)
***Psiloglonium macrosporum***	GMB5909	PZ281534	PZ332763	PZ513298	PZ544648	NA	This study
***Psiloglonium macrosporum***	GMB5910	PZ281535	PZ332764	PZ513299	PZ544649	NA	This study
*Psiloglonium macrosporum*	MFLUCC 13-0448^T^	KU243048	KU243049	NA	NA	NA	Li et al. (2016)
*Psiloglonium macrosporum*	MFLU 16-0955	NA	MH535903	NA	NA	NA	Jayasiri et al. (2018)
*Psiloglonium macrosporum*	MFLU 18-2218	OR225075	OP612525	OR134446	OR140436	NA	Zhang et al. (2023)
*Psiloglonium multiseptatum*	MFLUCC 11 C0164	NA	KP744512	NA	NA	NA	Liu et al. (2015)
*Psiloglonium sasicola*	MFLUCC 10-0565	KP744467	KP744513	NA	NA	NA	Liu et al. (2015)
*Psiloglonium simulans*	CBS:206.34	NA	FJ161178	FJ161139	FJ161094	FJ161116	Boehm et al. (2009b)
*Psiloglonium simulans*	ANM1557	NA	GQ221873	NA	NA	NA	Mugambi and Huhndorf (2009)
***Psiloglonium yunnanense***	GMB5907^T^	PZ281532	PZ332765	PZ513296	PZ544646	NA	This study
***Psiloglonium yunnanense***	GMB5908	PZ281533	PZ332766	PZ513297	PZ544647	NA	This study
*Rhytidhysteron bannaense*	KUMCC21-0482^T^	OP526398	OP526408	OP526395	OP572199	NA	Du et al. (2023)
*Rhytidhysteron bruguierae*	MFLU18-0571^T^	NA	MN017833	MN017901	MN077056	NA	Dayarathne et al. (2020)
*Rhytidhysteron camporesii*	KUN HKAS104277^T^	MN429069	MN429072	NA	MN442087	NA	Ekanayaka et al. (2020)
*Rhytidhysteron chromolaenae*	MFLUCC17 1516^T^	MN632461	MN632456	MN632467	MN635663	NA	Mapook et al. (2020)
*Rhytidhysteron coffeae*	KUMCC21 0489^T^	OP605963	OP526406	OP526412	OP572201	NA	Du et al. (2023)
*Rhytidhysteron erioi*	MFLU16-0584^T^	MN429068	MN429071	NA	MN442086	NA	Hyde et al. (2020)
*Rhytidhysteron magnoliae*	MFLUCC18 0719^T^	MN989383	MN989384	MN989382	MN997309	NA	De Silva et al. (2020)
*Rhytidhysteron mangrovei*	MFLU18 1894^T^	MK425188	MK357777	NA	MK450030	NA	Kumar et al. (2019)
*Rhytidhysteron mengziense*	KUMCC21 0490^T^	OP526402	OP526396	OP526414	OP572203	NA	Du et al. (2023)
*Rhytidhysteron mexicanum*	RV17107.1^T^	MT626026	MT626028	NA	NA	NA	Cobos-Villagrán et al. (2021)
*Rhytidhysteron neorufulum*	MFLUCC13-0216^T^	KU377561	KU377566	KU377571	KU510400	NA	Thambugala et al. (2016)
*Rhytidhysteron rufulum*	MFLUCC14-0577^T^	KU377560	KU377565	KU377570	KU510399	NA	Thambugala et al. (2016)
*Rhytidhysteron tectonae*	MFLUCC13-0710^T^	KU144936	KU764698	KU712457	KU872760	NA	Doilom et al. (2017)
*Rhytidhysteron thailandicum*	MFLUCC14-0503^T^	KU377559	KU377564	KU377569	KU497490	NA	Thambugala et al. (2016)
*Rhytidhysteron xiaokongense*	KUMCC20-0160^T^	MZ346022	MZ346012	MZ346017	MZ356246	NA	Ren et al. (2022)
*Rhytidhysteron yunnanense*	KUMCC21-0485^T^	OP526410	OP526404	OP526400	OP572205	NA	Du et al. (2023)

Type specimens are marked with “^T^”; “NA” indicates no sequence available in GenBank; newly generated sequences are indicated in bold.

###### Type.

China • Yunnan Province, Ailaoshan (24°1'16.35"N, 101°32'36.31"E), altitude: 1778 m, on decaying bamboo (*Poaceae*), 17 October 2024, Qing Yang 2024ALS227 (holotype; GMB5907) • *ibid*., isotype: KUN-HKAS151617.

###### Description.

Saprobic on decaying bamboo. ***Sexual morph*: *Hysterothecia*** 615–1026 µm long, 295–476 µm wide, 260–573 µm high (x̄ = 846 × 391 × 437 μm, n = 10), solitary to gregarious, irregularly scattered, superficial, dark, short to elongate, or irregular, with central, slit-like opening. ***Peridium*** 35–64 μm wide, thick-walled, of unequal thickness, poorly developed at the base, slightly thick at sides, carbonaceous, opaque dark and glossy. ***Hamathecium*** composed of numerous, dense, trabeculate pseudoparaphyses, 0.7–1.2 μm wide, anastomosing, embedded in a hyaline to pale brown gelatinous matrix, slightly enlarged at the upper ends. ***Asci*** 95–122.5 × 14.3–18.1 μm (x̄ = 108.9 × 16.1 μm, n = 25), 8-spored, bitunicate, fissitunicate, cylindrical to cylindric-clavate, short pedicellate with a knob-like pedicel, apically rounded with well-developed ocular chamber, easily broken in external layer. ***Ascospores*** 31.5–39 × 7–9.5 μm (x̄ = 35.8 × 8.2 μm, n=30), overlapping 1–2-seriate, fusiform, tapering toward ends, hyaline to subhyaline, 1-septate, constricted at the septum, upper cell larger than lower cell, smooth-walled, with small and large guttules. ***Asexual morph***: Undetermined.

**Figure 3.**
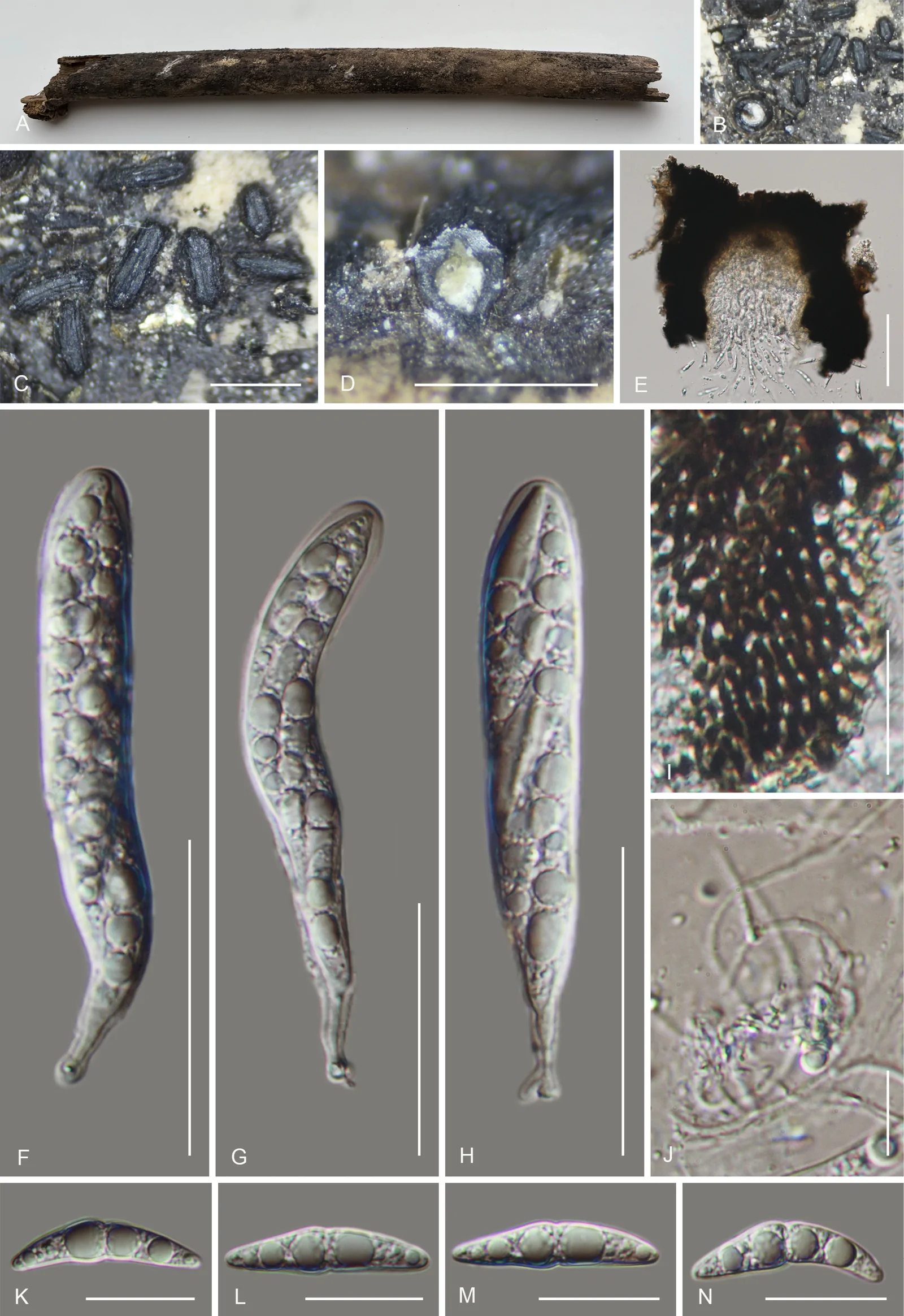
*Psiloglonium yunnanense* (GMB5907, holotype). **A**. Bamboo host substrate; **B, C**. Hysterothecia on host surface; **D**. Longitudinal sections through hysterothecium **E**. Vertical section of hysterothecium; **F–H**. Asci; **I**. Peridium; **J**. Hamathecium; **K–N**. Ascospores. Scale bars: 1 mm **(C, D)**; 100 μm **(E)**; 50 μm **(F–H)**; 10 μm **(J)**; 20 μm **(I, K–N)**.

###### Paratype.

China • Yunnan Province, Ailaoshan (24°5'6.21"N, 101°31'29.42"E), altitude: 1195 m, on decaying bamboo (*Poaceae*), 17 October 2024, Qing Yang, 2024ALS290 (GMB5908).

###### Notes.

Phylogenetic analysis (Fig. 1) based on ITS, LSU, SSU, *tef*1-α and *rpb*2 sequences revealed that our new collection *Psiloglonium yunnanense* is closely related to *P. sasicola* forming a sister species relationship (100/1.00; ML/BI). The LSU and ITS sequences of *Psiloglonium yunnanense* differ from those of *P. sasicola* (MFLUCC 10-0565) by 8/510 bp (1.6%, without gaps) and 30/482 bp (6.2%, 4 with gaps), respectively. *Psiloglonium yunnanense* is morphologically similar to *P. sasicola* (MFLUCC 10-0565), but can be distinguished by its larger hysterothecia (260–573 × 295–476 μm vs. 130–180 × 165–210 μm), larger asci (95–122.5 × 14.3–18.1 μm, x̄ = 108.9 × 16.1 μm vs. 80–105 × 13–15 μm, x̄ = 92 × 14.3 μm), significantly larger ascospores (31.5–39 × 7–9.5 μm, x̄ = 35.8 × 8.2 μm vs. 27–30 × 6–7 μm, x̄ = 28.3 × 6.8 μm), and the absence of a wide mucilaginous sheath surrounding the ascospores (present in *P. sasicola*) (Liu et al. 2015). In addition, *P. yunnanense* also resembles *P. ephedrae*, which similarly exhibits relatively large ascospores. *Psiloglonium ephedrae* differs in having wider ascospores (26–35 × 8–15 μm vs. 31.5–39 × 7–9.5 μm in *P. yunnanense*), with the upper cells being broadly ovate. Moreover, *P. ephedrae* was collected from *Ephedra andicola* (Boehm et al. 2009a), whereas our new collection was found on bamboo host. Based on these distinct morphological differences and phylogenetic separation, *P. yunnanense* is introduced here as a new species.

## Discussion

Morphological and ecological characteristics serve as fundamental criteria for species delimitation of *Ericboehmia* and *Psiloglonium*, but the relative importance of these traits differs between the genera (Boehm et al. 2009a, 2009b; Gardiennet et al. 2019; Raymundo et al. 2024). Within *Ericboehmia*, morphological characters, especially ascospore features, are key to species delimitation. Species within the genus can be readily distinguished based on ascospore length (ranging from 22–150 μm) (Barr 1975; Tibpromma et al. 2017; Jayasiri et al. 2018; Gardiennet et al. 2019; Raymundo et al. 2024), spore color, secondary septation patterns, presence of appendages, and ascomata dimensions (Gardiennet et al. 2019; Raymundo et al. 2024). The newly described *E. guizhouensis* conforms to this pattern, being clearly distinguishable from all previously described species by its comparatively short asci and ascospores.

Ecologically, *Ericboehmia* species have been reported as saprobes on woody plants, occurring across a wide range of hosts and habitats in both tropical and temperate forests (Barr 1975; Jayasiri et al. 2018; Gardiennet et al. 2019; Raymundo et al. 2024). For example, *E. saulensis* (the type species) occurs on dead *Caesalpinia* twigs (*Leguminosae*) in French Guiana, while several Asian species (*E. centramura*, *E. doimaeensis*, and *E. thailandica*) have been found on decaying wood in Thailand (Tibpromma et al. 2017; Jayasiri et al. 2018). *Ericboehmia beejakoshae* was collected from thorny twigs in the Andaman Islands (Niranjan and Sarma 2018). In temperate regions, *E. appendiculata* grows on *Nothofagus* bark in Patagonia (Sánchez et al. 2018), and *E. curtisii* has been reported from grapevine (*Vitis*) in the southeastern USA (Barr 1975). Prior to this study, *Ericboehmia* had not been documented from China. The discovery of *E. guizhouensis* in the present study from dead twigs in Guizhou Province extends the known Asian distribution of the genus and represents its first record in China.

The genus *Psiloglonium* demonstrates high ecological plasticity, with species colonizing a broad spectrum of angiosperm hosts and substrates across four continents (Boehm et al. 2009a, 2009b; Mugambi and Huhndorf 2009; Schoch et al. 2009; Liu et al. 2015; Li et al. 2016; Jayasiri et al. 2018; Zhang et al. 2023; Sun et al. 2025). This diversity in host association highlights the adaptive versatility of the genus. *Psiloglonium araucanum* is known on rotten branches of *Lardizabala (Lardizabalaceae)* in South America (Chile) (Boehm et al. 2009a), while *P. chambianum* occurs on wood of *Lonicera implexa* (*Caprifoliaceae*) in Africa (Tunisia) (Boehm et al. 2009a). In Central America, *P. clavisporum* has been found on decorticated wood in Nicaragua, whereas South American diversity includes *P. colihuae* on stems of *Chusquea culeou* (*Poaceae*) in Argentina, *P. ephedrae* on dead branches of *Ephedra andina* (*Ephedraceae*) in Chile, and *P. hysterinum* on bark in Brazil (Boehm et al. 2009a; Mugambi and Huhndorf 2009; Schoch et al. 2009; Liu et al. 2015). African representatives further include *P. compactum* on seeds of *Strychnos aculeata* (*Loganiaceae*) in Guinea. Asian species are represented by *P. macrosporum* and *P. multiseptatum* on unidentified twigs and *Thysanolaena maxima* (*Poaceae*) stems, respectively, in Thailand, and *P. sasicola* on dead culms of *Sasa (Poaceae)* in Japan (Boehm et al. 2009a; Liu et al. 2015; Li et al. 2016; Jayasiri et al. 2018; Zhang et al. 2023). In Europe, *P. pusillum* colonizes twigs of *Juniperus phoenicea* (*Cupressaceae*) in France, and *P. ruthenicum* occurs on *Quercus robur* (*Fagaceae*) in Ukraine. North American collections include *P. simulans* on wood in New York and *P. stygium* on bark in Pennsylvania, while *P. uspallatense* is known from dead branches of *Bulnesia retama* (*Zygophyllaceae*) in Argentina (Boehm et al. 2009a, 2009b; Rossman et al. 2016).

Phylogenetically, our newly described *Psiloglonium yunnanense* clustered closely to *P. sasicola*, a species reported on bamboo (*Poaceae*) from Thailand (Liu et al. 2015). This close relationship, coupled with the shared affinity for poaceous hosts, suggests that host substrate preference may be a phylogenetically conserved trait within this lineage. Such host specificity may reflect evolutionary adaptation to particular plant groups, especially within *Poaceae*. From China, three species of the genus have previously been reported, *P. bambusicola*, *P. brunneum*, and *P. guizhouense*, all of which are associated with poaceous hosts. This pattern supports the hypothesis that members of *Psiloglonium* in this region show a strong ecological preference for poaceous hosts (Boehm et al. 2009a, 2009b; Mugambi and Huhndorf 2009; Schoch et al. 2009; Liu et al. 2015; Li et al. 2016; Jayasiri et al. 2018; Zhang et al. 2023; Sun et al. 2025).

The report of two new species in this study highlights the still underexplored microfungal diversity of southwestern China and emphasizes the importance of targeted sampling in subtropical ecosystems, which are often rich in undocumented fungal taxa (Feng and Yang 2018; Zhang et al. 2020, 2023; Dissanayake et al. 2024; Habib et al. 2025; Liu et al. 2025; Yang et al. 2025). With only a limited number of *Ericboehmia* and *Psiloglonium* taxa previously recorded from the region, these additions increase the known species richness of *Hysteriaceae* in Asia and underscore the value of continued surveys on woody litter in biodiversity hotspots. Continued exploration is likely to reveal additional novel taxa and contribute to a more comprehensive understanding of fungal diversity and biogeography in the region.

## Supplementary Material

XML Treatment for Ericboehmia guizhouensis

XML Treatment for Psiloglonium macrosporum

XML Treatment for Psiloglonium yunnanense

## Additional information

### Artificial Intelligence (AI) use

The authors accept full responsibility for the content of the manuscript, including the disclosure of any use of AI.

No AI tools were used in the preparation of this manuscript.

### Author ORCIDs

Q. Yang https://orcid.org/0009-0006-2794-5583

Y.-L. Ren https://orcid.org/0009-0003-9063-425X

L.-L. Liu https://orcid.org/0009-0003-8481-6137

S.-P. Zou https://orcid.org/0009-0001-8515-6885

K. Habib https://orcid.org/0000-0003-2572-0306

S. Boamah https://orcid.org/0000-0003-0243-7276

N.N. Wijayawardene https://orcid.org/0000-0003-0522-5498

A.M. Elgorban https://orcid.org/0000-0003-3664-7853

F.Z. Filimban https://orcid.org/0000-0001-7527-1313

R. Cheewangkoon https://orcid.org/0000-0001-8576-3696

Q.-R. Li https://orcid.org/0000-0001-8735-2890

### Data availability

All of the data that support the findings of this study are available in the main text.
